# Disinfectant Susceptibility of Biofilm Formed by *Listeria monocytogenes* under Selected Environmental Conditions

**DOI:** 10.3390/microorganisms7090280

**Published:** 2019-08-21

**Authors:** Krzysztof Skowron, Ewa Wałecka-Zacharska, Katarzyna Grudlewska, Piotr Gajewski, Natalia Wiktorczyk, Magdalena Wietlicka-Piszcz, Andżelika Dudek, Karolina Jadwiga Skowron, Eugenia Gospodarek-Komkowska

**Affiliations:** 1 Department of Microbiology, Nicolaus Copernicus University in Toruń, L. Rydygier Collegium Medicum in Bydgoszcz, 9 M. Skłodowska-Curie St., 85-094 Bydgoszcz, Poland; 2 Department of Food Hygiene and Consumer Health, Wrocław University of Environmental and Life Sciences, 31 C.K. Norwida St., 50-375 Wrocław, Poland; 3 Department of Theoretical Foundations of Biomedical Sciences and Medical Computer Science, Nicolaus Copernicus University in Toruń, L. Rydygier Collegium Medicum in Bydgoszcz, 9 M. Skłodowska-Curie St., 85-094 Bydgoszcz, Poland; 4 Faculty of Telecommunication, Information Technology and Electrical Engineering, Institute of Telecommunications and Computer Science, UTP University of Science and Technology, Al. prof. S. Kaliskiego 7, 85-796 Bydgoszcz, Poland

**Keywords:** *L. monocytogenes*, biofilm formation, biofilm resistance, disinfectants, stainless steel, environmental conditions

## Abstract

*Listeria monocytogenes* is a one of the most important food-borne pathogens. Its ability to form biofilm contributes to increased resistance to disinfectants and inefficient disinfection, posing a serious threat for the food industry, and in the end the consumer. The aim of this study was the comparison of the biofilm formation ability of *L. monocytogenes* strains on stainless steel, under different environmental conditions (temperature, pH, NaCl concentration, nutrients availability), and the assessment of biofilm susceptibility to disinfectants. The bactericidal activity of four disinfectants in two concentrations (100% and 50% of working solution) against biofilm was conducted on four clinical strains, four strains isolated from food and one reference strain ATCC 19111. It was found that biofilm susceptibility to disinfectants was influenced by environmental conditions. Biofilm susceptibility correlated with the decrease of temperature, pH, nutrients availability and salinity of the environment. The least sensitive to disinfectants was biofilm produced at pH = 4 (the bacterial number ranged from 0.25 log CFU × cm^−2^ to 1.72 log CFU × cm^−2^) whereas the most sensitive was biofilm produced at pH = 9 (5.16 log CFU × cm^−2^ to 7.84 log CFU × cm^−2^). Quatosept was the most effective disinfectant, regardless of the conditions. In conclusion, biofilm susceptibility to disinfectants is strain-dependent and is affected by environmental conditions.

## 1. Introduction

*Listeria monocytogenes* is a Gram-positive, non-spore forming, rod-shaped bacterium, widespread in the environment. The major source of human infections is food, especially RTE (ready-to-eat) food not requiring heating before consumption [1,2]. In 2017, 2480 confirmed cases of invasive listeriosis, including 227 deaths, were reported in the European Union [3]. The most frequently implicated in infected RTE food were fish products (6.2%) and meat products (2.5%) [3]. 

One of the major causes of food contamination with *L. monocytogenes* is its ability to form biofilm and to survive under adverse environmental conditions [4,5]. Biofilm is a self-regulating, integrated, thin structure made up of one or more species of bacteria, encased in a self-produced extracellular matrix [4]. Bacteria of the *Listeria* genus are capable of producing biofilm on various surfaces, including polypropylene, stainless steel and glass. The ability to produce biofilm depends on many factors: the type of surface, the type and physiological state of bacteria, the existence of other biofilms on a given surface, etc., [6]. It has been shown that the presence of by-products during food production, including meat juice, pork serum and/or fat, stimulates the formation of biofilms [7]. The multilayered structure of biofilm facilitates access to nutrients and the removal of metabolites, while it hinders access of biocides to deeper biofilm layers [8]. Bacteria in such a community are much more resistant to stress conditions, antimicrobials and disinfectants compared with the planktonic cells [9]. Temporary lack of hygiene or ineffective disinfection in the food processing facility enable colonization of working surfaces by the pathogen and it’s transmission to the food [4,10]. *L. monocytogenes* was detected on food-processing equipment (gasket, knives, tables, cutting machines, conveyor belts) as well as on floors and walls [10]. The most frequently used disinfectants include chlorine-based disinfectants, iodophors, oxidizing agents, alcohols, surfactants, quaternary ammonium compounds, organic and inorganic acids [11]. Biofilm eradication efficacy based on cleaning and disinfection depends on complexity and thickness of biofilm layers and the surface type. It has been shown that stainless steel is non-porous and corrosion-resistant, and that the cleanability and disinfectability of such surfaces are significantly better than polymers [12]. Bacteria adhere more quickly to damaged surfaces, i.e., scratches and crevices, in which water and nutrients accumulate. In addition, bacteria in these niches are protected against cleaning and disinfection [10].

The study of the influence of environmental factors on the biofilm formation by *L. monocytogenes* has been presented by many authors. However, these studies were carried out in polystyrene titration plates that assessed the impact of up to two stress factors on biofilm formation [13,14,15,16]. There is also little data on the effect of biofilm formation conditions on its subsequent resistance to disinfection. Therefore, the aim of the present study is to assess biofilm formation of *L. monocytogenes* strains under four stress conditions, most frequently encountered by the pathogen in the food processing environment (temperature, pH, salinity and nutrient availability). Additionally, susceptibility of biofilm formed under different environmental conditions to selected disinfectants is determined. The selection of these conditions, i.e., the temperature, pH, salinity and availability of nutrients, are correlated with the conditions that bacteria may encounter in the food industry environment. In practice, *L. monocytogenes* biofilms may be formed both under refrigeration conditions and, for example, on smoking trolleys. For this reason, it is reasonable to check the effect of temperature on the biofilm formation and its resistance to disinfectants. Likewise, *L. monocytogenes* can form biofilms on the elements of machines injecting meat and fish with brine and on surfaces contacted with pickled food products. This justifies including in the research model different levels of salinity and the pH of the environment. Also, the level of organic pollution of the surfaces on which the biofilm is formed may be different, which, in turn, determines the availability of additional nutrients sources, which we have also included in our research. The values of the examined environmental conditions have been selected on the basis of the actual conditions in food processing plants and information from the references on the minimum and maximum values of factors tolerated by *L. monocytogenes*. The literature provides data on increased resistance of *L. monocytogenes* in the biofilm structure to a number of disinfectants. Nevertheless, our research allows us to assess the resistance to selected disinfectants against biofilms, which have formed under changing environmental conditions.

## 2. Results

### 2.1. Assessment of Biofilm Formation Ability under Different Environmental Conditions

It was found that biofilm formation ability, to a certain extent, was strain-dependent and was affected by environmental conditions. The strongest biofilm was formed in the alkaline environment (pH = 9) and in the environment of decreased nutrients availability (0.5 BHI—Brain Heart Infusion), whereas the weakest biofilm was found in the presence of 10% NaCl. The number of bacteria recovered from biofilm ranged from 3.86 log CFU × cm^−2^ (ATTC, 10% NaCl) to 7.9 log CFU × cm^−2^ (6F, 0.5 BHI). 

Statistical analysis showed that the number of bacteria recovered from the biofilm was not dependent on the strain, and that the differences found in the number of bacteria recovered from the biofilm resulting from different environmental conditions for individual *L. monocytogenes* strains tested were not statistically significant (Table 1). Therefore, the strain effect on biofilm formation was omitted in further analyses.

**Figure 1:**
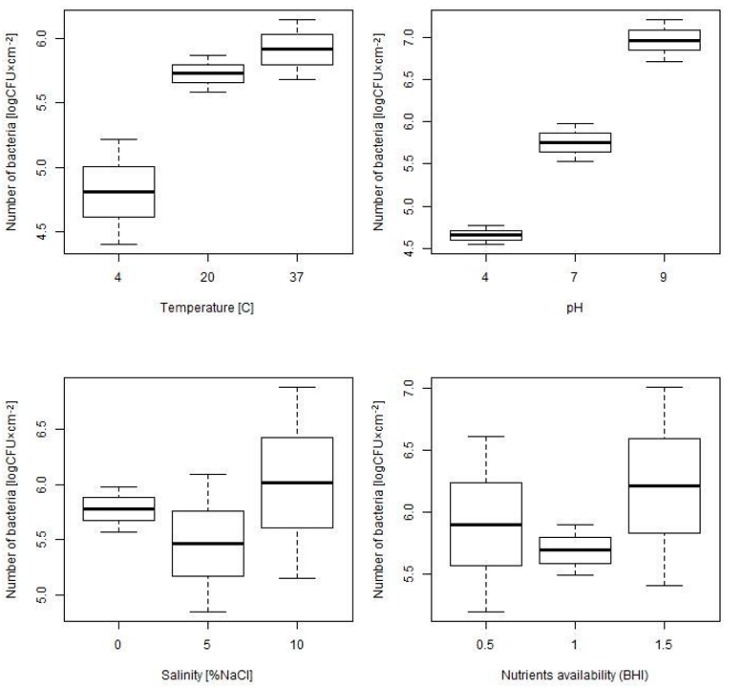
The distribution of the number of bacteria recovered from biofilm formed under various environmental conditions—the boxplots show mean with SE (Standard error) and 95% confidence interval.

The distribution of bacteria number recovered from biofilm formed under different environmental condition, summarized for all tested strains, is presented at Figure 1.

In Table 2, the number of bacteria recovered from biofilm formed under various environmental conditions, is summarized. The obtained results indicate that the number of bacteria from biofilm significantly depends on the temperature and pH. The increase of the temperature is associated with the increase in bacteria number; for the temperature 4 °C, 20 °C and 37 °C the mean (SD) numbers of bacteria were as follows: 4.81 (0.83), 5.73 (0.29) and 5.91 (1.33), *p* = 0.001, respectively. The increase of pH is also associated with the increase of bacteria number and for subsequent pH values equal to 4, 7 and 9 the number of bacteria increased from 4.66 (0.23) for pH = 4, 5.76 (1.26) for pH = 7 to 5.91 (1.33) for pH = 9, *p* < 0.001. The performed analysis shows that the salinity and nutrients availability have no significant effect on biofilm formation, although in the study population a small variability was observed for these factors (Table 2, Figure 1).

To study the association between the number of bacteria in biofilm and the considered environmental conditions such as temperature, pH, salinity and nutrient availability, multiple regression analysis has been used. Table 3 contains the estimates of the final model. The temperature and the pH were included as significant covariates in the model. The temperature of 4 °C is associated with a lower number of bacteria (on average lower by 1.15 log CFU × cm^−2^, *p* < 0.001) as compared to a temperature of 37 °C, while for the temperatures 20 °C and 37 °C, no significant difference in bacteria number was found. The pH value of 4 is associated with a smaller number of bacteria, as compared to the pH of 7 (−1.3 log CFU × cm^−2^, *p* < 0.001), while pH = 9 is associated with a larger number of bacteria (1.01 log CFU × cm^−2^, *p* < *0*.001) as compared to pH = 7. No significant association was found between the number of bacteria and the salinity and nutrients availability.

### 2.2. Assessment of Disinfectant Susceptibility of Biofilm Formed under Different Environmental Conditions

It was found that for the biofilm produced at 4 °C, the reduction of bacteria number ranged from 0.85 log CFU × cm^−2^ (6F, 50.0% working solution of Peroxat) to 4.64 log CFU × cm^−2^ (ATCC 19111, Quatosept and Chlorox S, concentration 100.0%). At 20 °C, the reduction of bacteria number ranged from 1.79 log CFU × cm^−2^ (6F, 50.0% Peroxat) to 5.83 log CFU × cm^−2^ (2B, 100.0% Quatosept and Chlorox S), whereas at 37 °C, it ranged from 2.90 log CFU × cm^−2^ (5F, 50.0% Peroxat) to 6.36 log CFU × cm^−2^ (2B, 100.0% Chlorox S and Quatosept).

For the biofilm formed at pH = 4, the reduction number of bacteria ranged from 0.25 log CFU × cm^−2^ (6F, Jodat, 50.0%) to 1.72 log CFU × cm^−2^ (ATCC 19111, Quatosept, 100.0%). At pH = 7, the reduction of bacteria number ranged from 2.85 log CFU × cm^−2^ (6F, Peroxat and Jodat, 50.0%) to 6.36 log CFU × cm^−2^ (2B, Chlorox S and Quatosept, 100.0%) while at pH = 9 ranged from 5.16 log CFU × cm^−2^ (8D, Peroxat, 50.0%) to 7.84 log CFU × cm^−2^ (8D, Jodat, Chlorox S and Quatosept, 100.0%). 

The reduction of bacteria number for biofilm created at 0% NaCl ranged from 2.85 log CFU × cm^−2^ (6F, Peroxat and Jodat, 50.0%) to 6.28 log CFU × cm^−2^ (8D, Chlorox S and Quatosept, 100.0%). In the environment of 5 and 10% NaCl the reduction of bacteria number ranged from 1.38 log CFU × cm^−2^ (6F, Peroxat, 50.0%) to 4.40 log CFU × cm^−2^ (ATCC 19111, Quatosept, 100.0%) and from 0.26 log CFU × cm^−2^ (5F, Peroxat, 50.0%) to 2.71 log CFU × cm^−2^ (ATCC 19111, Quatosept, 100.0%), respectively.

In Table 4, the reductions of bacteria number reisolated from biofilm, for various disinfectants, are summarized. The highest reduction of bacteria number was achieved by the use of Quatosept (mean (SD) = 3.71 (1.84)) and the obtained decrease in bacteria number was significantly higher than that obtained by the use of Jodat and Peroxat, where the mean (SD) reduction was respectively 3.04 (1.91) and 3 (1.89), *p* < 0.001. The next disinfectant was Chlorox S (mean (SD) = 3.43 (1.91)), for which a significantly higher reduction of bacteria number was obtained, in comparison to Jodat (3.04 (1.91), *p* = 0.012) and Peroxat (3 (1.89), *p* = 0.006).

The lowest reduction number of bacteria was calculated for the bacteria incubated in a medium of decreased nutrients availability (0.5 BHI) and ranged from 0.89 log CFU × cm^−2^ (5F, Jodat, 50.0%) to 3.53 log CFU × cm^−2^ (ATCC 19111, Quatosept, 100.0%) (Table 5). For the biofilm formed at standard conditions (1.0 BHI) the reduction of bacteria number ranged from 3.85 log CFU × cm^−2^ (5F, Peroxat and Jodat, 50.0%) to 6.28 log CFU × cm^−2^ (8D, Chlorox S and Quatosept, 100.0%) whereas in the medium with increased nutrients (1.5 BHI), it ranged from 2.42 log CFU × cm−2 (4-CSF, Jodat, 50.0%) to 5.36 log CFU × cm^−2^ (6F, Quatosept, 100.0%).

The reductions of bacteria number for the two considered concentrations of the disinfectants are summarized in Table 5. The higher disinfectant concentration is associated with higher reduction of bacteria number. For concentration 50% and 100%, the mean (SD) reduction of bacteria number is respectively equal to 2.81 (1.83) and 3.78 (1.87), *p* < 0.001.

**Figure 2:**
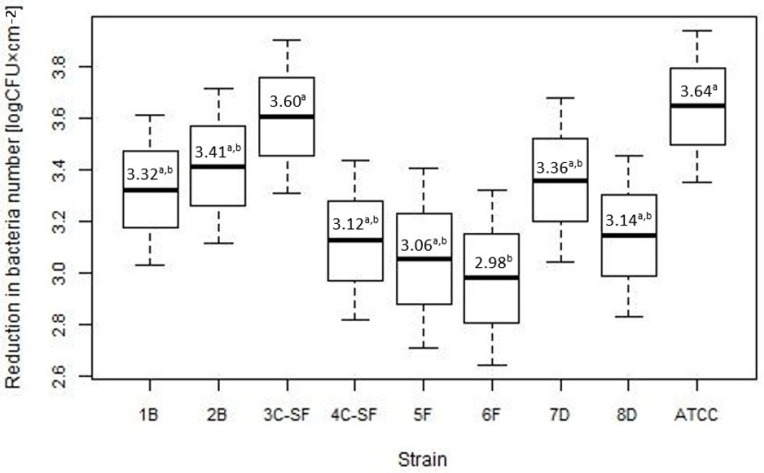
Reductions of bacteria numbers reisolated from biofilm for various strains—the boxplots show mean with SE and 95% confidence interval (a, b, c: differences between values marked with different letters are statistically significant). 1B, 2B—strains from blood, 3C-SF, 4C-SF—strains from cerebrospinal fluid, strains isolated from food—2 from fish: 5F, 6F and 2 from dairy products: 7D, 8D, ATCC 19111—reference strain.

The analysis of the reductions of bacteria numbers for various strains has also been performed and the results show that there is the significant difference (*p* = 0.024) between the reductions for various strains. Multiple comparisons performed have shown that a statistically significant difference exists only between the decrease in the number of bacteria found for the 6F strain and the 3 C-SF and ATCC strains (Figure 2). The distribution of the reduction numbers for various strains is shown in Figure 2.

**Figure 3:**
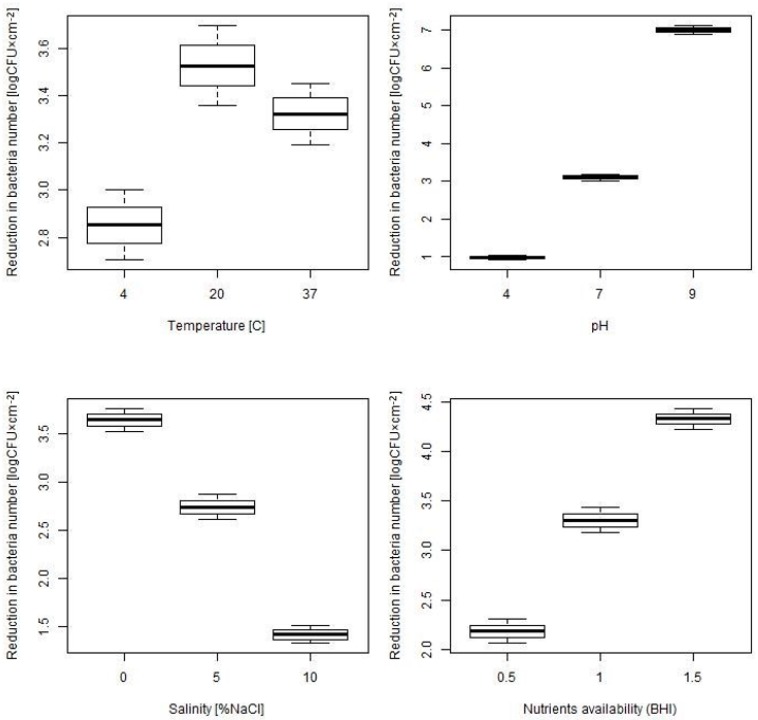
Resistance to disinfection of biofilm formed under different environmental conditions, expressed as decreases in the number of bacteria isolated from the biofilm—the boxplots for mean with SE (standard error) and 95% confidence interval.

The distribution of the decreases in bacteria numbers recovered from biofilm formed under different environmental conditions, summarized for all tested strains, disinfectants and its concentrations, is presented in Figure 3.

The statistical analysis carried out showed that each of the considered environmental conditions statistically influenced the biofilm resistance to disinfectants, expressed as decreases in the number of *L*. *monocytogenes* recovered from the biofilm (Table 6). In the case of temperature, the recorded decrease in the number of bacteria recovered from the biofilm (mean (SD)), ranged from 2.85 (0.9) log CFU × cm^−2^ at 4 °C to 3.53 (1.03) log CFU × cm^−2^ at 20 °C (Table 6). This means that the biofilm formed at 4 °C was the most resistant to disinfection and the one most susceptible was formed at 20 °C. Resistance differences between biofilm formed at 4 °C and 20 °C were statistically significant (Table 6). In the case of pH, there was a clear trend indicating that the more alkaline the environment in which a biofilm is formed, the lower its resistance to disinfection (Table 6). Decreases in the number of bacteria (mean (SD)) recovered from biofilms after disinfection ranged from 0.96 (0.38) log CFU × cm^−2^ at pH = 4 to 7.01 (0.73) log CFU × cm^−2^ at pH = 9. Differences in biofilm resistance to disinfection for all tested pH variants were statistically significant (Table 6). Similarly, the biofilm resistance to disinfection was influenced by the availability of nutrients in the environment in which the biofilm was formed (Table 6). It was shown that with higher nutrient availability, the biofilm was more sensitive to disinfection. The lowest decrease in the number of bacteria (mean (SD)) isolated from the biofilm after disinfection was found for 0.5 BHI—2.18 (0.75) log CFU × cm^−2^, and the highest for 1.5 BHI—4.32 (0.65) log CFU × cm^−2^. Differences in biofilm resistance to disinfection for all tested nutrient availability variants were significant statistically (Table 6). The opposite tendency was demonstrated for the influence of environmental salinity on the resistance of the formed biofilm to disinfection (Table 6). The lowest decrease in the number of bacteria (mean (SD)) isolated from the biofilm subjected to disinfection was found in the case of biofilms formed by salinity 10%—1.42 (0.55) log CFU × cm^−2^, and the highest at salinity 0%—3.64 (1.97) log CFU × cm^−2^. Differences in biofilm resistance to disinfection for all tested salinity variants were statistically significant (Table 6).

In order to analyze the association between the reduction of bacteria in biofilm and the applied disinfectants, concentration of the disinfectants and considered environmental conditions such as temperature, pH, salinity and nutrient availability, the linear mixed effects model has been fitted to the data. Table 7 contains the estimates of the final model. The adopted model unambiguously confirmed the results of analyses carried out using the decrease in the number of bacteria isolated from the biofilm. It has been shown that all tested factors, statistically, significantly affect the biofilm resistance to disinfection. Quatosept was found to be the most effective disinfectant, and the use of another of the tested compounds caused weaker eradication of biofilm, as evidenced by the recorded decreases in the number of bacteria isolated from the biofilm by 0.274–0.712 log CFU × cm^−2^, as compared to Quatosept (Table 7). It has also been shown that a higher concentration of each of the disinfected agents studied reduces the number of bacteria recovered from the biofilm more effectively, regardless of the strain and biofilm conditions (Table 7). The model also showed that the change in environmental conditions of biofilm formation compared to standard (for biofilm formation in the laboratory)—temperature 37 °C, pH = 7, salinity 0% NaCl and 1.0 BHI, resulted in a change of biofilm resistance to disinfection, as evidenced by changes in the decreases in the number of bacteria isolated from the biofilm. Based on the model, it was shown that the pH and salinity of the environment played the most important role in the experiment (Table 7).

## 3. Discussion

Since *L. monocytogenes* is able to survive at a wide range of pH, low temperatures and high salinity it may easily spread and survive in the environment. An important factor helping the pathogen to withstand deleterious conditions is biofilm formation ability. *Listeria* biofilms are a serious problem in the food industry, due to very fast adhesion (about 20 min) and maturity within 24 h. The structure of biofilm increases the chance of bacteria to survive at low and high temperatures and pH, high salinity and low nutrient availability, and protects the deeper layers of bacterial cells against disinfectants and antibiotics [8]. *Listeria* biofilms contribute to secondary food contamination posing risk to the public health [13,14]. In our research, we studied the biofilm formation ability of *L. monocytogenes* strains under different environmental conditions and assessed its susceptibility to disinfectants. We showed that temperature, pH and salinity of the environment and nutrient availability, all affect biofilm formation. It was found that intensity of biofilm formation increased together with temperature of the environment. This is in accordance with studies of Kadam et al. [15], Poimenidou et al. [16] and Bonsaglia et al. [17] who showed that higher temperature fostered biofilm formation. In contrast, Poimenidou et al. [18] reported more intensive biofilm formation at 20 °C than at 37 °C. This research, however, was conducted in polystyrene titration plates with a different culture medium than that which was used in our experiment. The lower biofilm levels at 37 °C could be attributed to possible detachment of cells during incubation rather than to reduced initial attachment.

The main role in maintaining homeostasis in bifilma is played by surface proteins, which play the role of a “linker” between the bacterial cell and the environment, and also play a key role in communication between cells, resistance to stress conditions and maintaining a balance between the amount of nutrients and toxins [19]. Numerous studies [20,21,22] on the *L*. *monocytogenes* genome have revealed the presence of 133 genes for surface proteins. However, there is still a knowledge gap regarding the comparison of subproteomic changes at different temperatures during biofilm formation [23]. The main carnitine transporter, OpuC, encoded by opuCABCD operon, is involved in the regulation of changes at the cellular level when *L*. *monocytogenes* is exposed to cold shock [24]. In turn, Santos et al. [25] evaluated protein synthesis by *L*. *monocytogenes* in three different temperature variants (10, 25 and 37 °C) and two phases of biofilm formation (early stage and mature biofilm). Santos et al. [25] showed that among 920 identified proteins, a significant number of them were associated with basic cellular functions, and some with thermoregulation. In addition, they showed that the role of ribosomes and stress proteins CtC and sigma B factor (σB) is significant in the adaptive mechanism for temperature changes during biofilm formation. In addition, the response to temperature stress during biofilm formation influenced changes in cell membrane fluidity and motility (higher PrsA2 and FlaA protein levels) and high overexpression of cold shock proteins, i.e., CspLA and DPS. In turn, at 37 °C, a higher level of gene transcription followed by translation of surface and stress proteins was observed compared to lower tested temperatures of biofilm formation [25].

In the present study the alkalization of the environment promoted biofilm formation whereas acidic pH decreased this ability. This is in agreement with the study of Tresse et al. [26] who found weaker biofilm formation at pH = 5 than at pH = 7. In turn, Harald and Zottola [27] proved that *L. monocytogenes* better form a biofilm when the environment in which they multiply is alkaline, than in an environment with pH < 7. Nilsson et al. [28] achieved different results, noting the strongest biofilm formation in an acidic environment. Nevertheless, they used a semi-quantitative crystal violet method, which measures total biomass within the biofilm including live, unculturable and dead cells, and possibly extracellular polymeric substances (EPS). In turn, Belessi et al. [29] showed no effect of pH of the environment on biofilm formation by *L. monocytogenes*. Belessi et al. [29], however, did not change medium and rinse coupons during incubation and as a result, metabolites e.g., lactates, produced by bacteria, lowered the pH of the medium.

The salinity of the environment also affected biofilm formation ability. We found that 5% NaCl additive in the medium promoted biofilm formation, as compared to a medium without salt, whereas 10% NaCl decreased this ability. This confirms results by Jensen et al. [13] who noticed increased biofilm formation in the presence of 5% NaCl, as compared to the medium without salt additive. This is, however, in contrast to a study of Xu et al. [30] who reported stronger biofilm formation at the concentration over 4% NaCl. Similar results were also obtained by Pan et al. [31], who in their study demonstrated that, among the sodium chloride concentrations ranging from 0.5 to 7% at 37 °C, the formation of biofilm was most strongly promoted by the concentration of 2% NaCl and the weakest by the concentration of 7% NaCl. Conversely, Lee et al. [32] did not observe any correlation between biofilm formation ability by *L. monocytogenes* and salinity of the environment. Nonetheless, this study was conducted only on one strain, ATCC 1912 [21]. Caly et al. [33] proved that there are no statistically significant differences between the number of biofilm forming bacteria in sodium chloride concentrations ranging from 0–6% NaCl. At the same time, they obtained a statistically significantly lower number of bacteria forming biofilms with a NaCl concentration of 11%.

In this study, we noted the strongest biofilm formation in a medium of limited nutrient content, which supports the studies of Kadm et al. [15] and Cherifi et al. [34]. On the contrary, Poimenidou et al. [18] and Zeraik and Nitschke [35] showed that rather rich nutrients content fosters biofilm formation ability. These differences might be explained by the strains and medium used. Folsom et al. [36] observed a relationship between the serotype of *L. monocytogenes* and their ability to produce biofilms under different nutrient availability conditions. They showed that strains belonging to serotype 4b more strongly formed biofilms under conditions of normal nutrient availability, whereas serotype 1/2a more strongly formed biofilms in the case of limiting their availability [36].

Among all of the disinfectants tested in our study, the most effective was Quatosept, whereas the lowest bactericidal activity displayed Jodat and Peroxat. This supports the study of Dhowlaghar et al. [37] who found that the most and the least efficient biofilm eradication agents are quaternary ammonium compounds and oxidizing agents, respectively. Also, Piercey et al. [38] demonstrated the high antilisterial activity of quaternary ammonium compounds (QAC), while Aarnisalo et al. [39] stated that QAC is less effective than chlorine-based agents, alcohols and peracetic acid.

In the present study it was shown that the efficacy of biofilm eradication was influenced by the conditions of biofilm formation. The most susceptible to disinfectants was biofilm produced at pH = 9, whereas the least sensitive was biofilm formed at pH = 4 or in the presence of 10% NaCl. This is not in agreement with the earlier studies of Nilsson et al. [28] and Kastbjerg and Gram [40]. Nilsson et al. [28] stated that biofilm sensitivity to QAC is not pH-dependent, whereas Kastbjerg and Gram [40] found that an increase of environment salinity positively correlates with biofilm susceptibility to oxidizing agents, but has no impact on tolerance to QAC. Similar results were obtained by Ren and Frank [41], demonstrating the high efficacy of benzalkonium chloride against the biofilm produced by *L. monocytogenes* at 21 °C. Kostaki et al. [42] showed that QACs and mixtures of peracetic acid and hydrogen peroxide act almost equally on biofilm formed by *L. monocytogenes* on the surface of steel coupons.

The discrepancy between our study and their studies might be explained by the growth conditions, media and surface tested. 

In this study, the biofilm susceptibility to disinfectants increased together with temperature. This is in accordance with results of Chaitiemwong et al. [43] who declared higher sensitivity at 37 °C than at 20 °C. In contrast, Olszewska et al. [44] proved greater efficacy of disinfection on biofilm formed at 15 °C rather than 37 °C. 

We also noted that the most resistant to disinfectants is biofilm produced in the environment of limited nutrients availability, which supports the study of Lee and Frank [45]. Research on the effect of nutrient availability on the sensitivity of biofilm produced by *L. monocytogenes* to disinfectants was also carried out by Kyoui et al. [46]. In their experiment, they used different concentrations of glucose (0.1%, 1%, 2%), and as a disinfectant, they used sodium hypochlorite. They showed that biofilm formed in conditions of increased availability of nutrients is characterized by greater resistance to disinfectants, which is different from the results obtained in this work. Ren and Frank [41] noted in their research that changing the availability of nutrients leads to changes in QAC resistance against *L. monocytogenes* planktonic cells, resulting in an increase in resistance as the amount of nutrients in the environment decreases.

On the contrary, Ren and Frank [41] showed no effect of nutrients availability on biofilm resistance to disinfectants. However, they used different media and growth conditions.

## 4. Materials and Methods 

### 4.1. Bacterial Strains 

The study was conducted on four clinical *L. monocytogenes* strains—2 from blood: 1B (serotype 1/2a-3a), 2B (serotype 1/2a-3a) and 2 from cerebrospinal fluid: 3C-SF (serotype 1/2a-3a), 4C-SF (serotype 4b-4d-4e) and four strains isolated from food—2 from fish: 5F (serotype 1/2a-3a), 6F (serotype 1/2b-3b) and 2 from dairy products: 7D (serotype 1/2c-3c), 8D (serotype 1/2a-3a) (collection of the Department of Microbiology, Nicolaus Copernicus University in Toruń, L. Rydygier Collegium Medicum in Bydgoszcz) and one reference strain ATCC 19111. All tested strains came from Poland and, except for the 6F strain, were sensitive to all antibiotics recommended in the European Committee on Antimicrobial Susceptibility Testing (EUCAST) Recommendations v.8.0 [47]. The 6F strain was resistant to penicillin, ampicillin and cotrimoxazole. Single colonies of bacteria were transferred onto Columbia Agar with 5% Sheep Blood (Becton Dickinson, Franklin Lakes NJ, United States) and incubated at 37 °C for 24 h. 

### 4.2. Biofilm Formation of L. monocytogenes Strains on Stainless Steel Coupons

Biofilm formation ability was assessed on stainless steel coupons (1 cm × 2 cm). Coupons were washed in the commercial detergent, soaked for 5 min in 70% ethanol (POCH) and autoclaved. Two coupons were placed in tubes containing 3 cm^3^ of suspension of each strain (0.5 Mc Farland scale) in Brain Heart Infusion Broth (BHI) (Merck, Warsaw, Poland) of selected parameters and were incubated for 72 h. The detailed model of the experiment together with the values of particular tested environmental parameters (variant of the experiment) are presented in Table 8. During incubation for each strain, except for variant 1, every 24 h medium was replaced with the fresh one and coupons were rinsed with sterile PBS (Phosphate Buffered Saline) (BTL, Warsaw, Poland). For variant 1 (4 °C) strain medium was replaced every 4 days and the incubation was extended to 12 days. As a negative control 2 coupons in a sterile BHI (Merck, Poland) of selected parameters were used.

### 4.3. Determination of Anti-Biofilm Efficacy of Selected Disinfectants

Effect of four disinfectants, in two working concentrations (50% and 100%), on biofilm was assessed (Table 9). Bacteria were grown on stainless coupons as described above and then coupons were placed in a tube containing 5 mL of disinfectant solution (1 coupon/one concentration of disinfectant). After 5 min coupons were rinsed with a sterile PBS (BTL, Poland) and neutralized in a water solution of Tween 80 (Sigma Aldrich, Saint Louis, MO, USA)—10 g, L-histidine (Sigma Aldrich)—0.5 g, lecithin (Sigma Aldrich)—1 g, Na_2_S2O_3_ (Avantor)—2.5 g. Next, coupons were rinsed again in 3 mL of PBS, sonicated (Ultrasonic DU-4, Nickel-Electro Ltd., Oldmixon, Great Britain) for 10 min at room temperature (30 kHz, 150 W) and shaken for 10 min at 400 rpm. After shaking 10-fold serial dilutions were made for obtained suspension of bacteria detached from coupons surface. Next, each dilution was plated onto Columbia Agar with 5% Sheep Blood (Becton Dickinson, Franklin Lakes NJ, United States) and incubated at 37 °C for 48 h. The number of grown bacteria colonies was calculated and converted to log CFU × cm^−2^ including dilution, diluent volume and coupon area. The positive control were bacteria growing on stainless steel coupon in BHI (Merck, Poland) of selected parameters not exposed to disinfectant but to hard water [48]. To assess anti-biofilm effectiveness of disinfectants, the reduction of bacteria number isolated from biofilm was calculated according to the formula:

R = K_WX_ − D_WX_

where R—reduction of *L. monocytogenes* number isolated from biofilm (log CFU × cm^−2^), K_WX_—number of control *L. monocytogenes* isolated from biofilm not exposed to disinfectants, D_WX_—number of *L. monocytogenes* isolated from biofilm exposed to disinfectants.

### 4.4. Statistical Analysis

The summary statistics for continuous variables are presented as mean and standard deviation (SD). Differences between continuous variables were analyzed by the t test for independent samples or by ANOVA together with the Benjamini–Hochberg type adjustment for multiple testing. To study the dependence between the number of bacteria in biofilm and the considered environmental factors, multiple linear regression analysis has been used. Initially, all considered environmental factors, i.e., the temperature, pH, salinity and the nutrient availability were included in the model as covariates. The backward elimination future selection procedure was applied to find the most significant subset of predictor variables.

To analyze the dependence between the reduction of the number of bacteria after the application of disinfectant and the environmental factors, the type and concentration of disinfectant, the Linear Mixed Effects Model (LMM) has been applied. Initially the considered environmental factors and the type of applied disinfectant and its concentration as well as interactions terms between the disinfectants types and environmental factors and interactions terms between the concentrations of the disinfectants and the environmental factors were included in the model as covariates. The strain was included as the random effects term. To select the most significant subset of predictor variables, the backward elimination future selection procedure was applied.

The results were considered as statistically significant when the *p*-value was less than 0.05. The statistical analysis was performed with the use of the R-software (packages lme4 and gls).

## 5. Conclusions

Disinfectant susceptibility is strain-dependent and is affected by the environmental conditions of biofilm formation. The presented results might be essential for the food industry by indicating the conditions where biofilm eradication is the most challenging and requires more effort. Biofilms are one of the major reasons for secondary food contamination in food processing plants. Therefore, there is a need for further studies on efficient disinfection to eliminate *L. monocytogenes* biofilm. 

## Figures and Tables

**Table 1:** Comparison of biofilm formation ability of *L. monocytogenes* strains for different strains.

Strain	*n*	Mean (log CFU × cm^−2^) (SD)	*p*-Value
1 B	18	5.71 (1.24)	0.161
2 B	18	5.73 (1.29)	
3 C-SF	18	5.85 (1.19)	
4 C-SF	18	5.55 (1.33)	
5 F	18	5.69 (1.33)	
6 F	18	6.28 (1.10)	
7 D	18	6.06 (1.14)	
8 D	18	5.77 (1.45)	
ATCC	18	5.28 (1.17)	

SD—standard deviation.

**Table 2:** Comparison of biofilm formation ability of *L. monocytogenes* strains under different environmental conditions.

Number of Bacteria (log CFU × cm^−2^)			*p*-Value			
Mean (SD)	Mean (SD)	Mean (SD)				
**Temperature**						
4 °C, *n* = 18	20 °C, *n* = 18	37 °C, *n* = 126	*p*	*p* 4–20	*p* 4–37	*p* 20–37
4.81 (0.83)	5.73 (0.29)	5.91 (1.33)	0.001	0.036	0.001	0.541
**pH**						
pH 4, *n* = 18	pH 7, *n* = 126	pH 9, *n* = 18	*p*	*p* 4–7	*p* 4–9	*p* 7–9
4.66 (0.23)	5.76 (1.26)	6.96 (0.5)	<0.001	<0.001	<0.001	<0.001
**Salinity**						
Salinity 0%, *n* = 126	Salinity 5%, *n* = 18	Salinity 10%, *n* = 18	*p*	*p* 0–5	*p* 0–10	*p* 5–10
5.78 (1.17)	5.47 (1.25)	6.02 (1.75)	0.744	/	/	/
**Nutrients Availability**						
Nutrients availability 0.5, *n* = 18	Nutrients availability 1, *n* = 126	Nutrients availability 1.5, *n* = 18	*p*	*p* 0.5–1	*p* 0.5–1.5	*p* 1–1.5
5.9 (1.44)	5.69 (1.16)	6.21 (1.62)	0.460	/	/	/

SD—standard deviation.

**Table 3:** Predictive factors for bacteria number in biofilm identified by linear model.

Factors	Value (log CFU × cm^−2^)	Standard Error	*t*-Value	*p*-Value
(Intercept)	5.95	0.12	49.1	<0.001
Temperature 4 vs. 37	−1.15	0.28	−4.12	<0.001
Temperature 20 vs. 37	−0.23	0.28	−0.82	0.414
pH 4 vs. 7	−1.3	0.28	−4.65	<0.001
pH 9 vs. 7	1.01	0.28	3.62	<0.001

**Table 4:** Reduction of bacteria number reisolated from biofilm for the investigated disinfectants.

Disinfectant	*n*	Mean (log CFU × cm^−2^) (SD)	Jodat	Peroxat	Chlorox S
			*p*-Value	*p*-Value	*p*-Value
Jodat	324	3.04 (1.91)			
Peroxat	324	3 (1.89)	0.772		
Chlorox S	324	3.43 (1.91)	0.012	0.006	
Quatosept	324	3.71 (1.84)	<0.001	<0.001	0.078

SD—standard deviation

**Table 5:** The reductions of bacteria numbers recovered from biofilm for the examined concentrations of disinfectants.

**Disinfectant Concentration**		***p*-Value**
**50% work solution**	**100% work solution**	
Mean reduction in bacteria number (SD)		
*n* = 648	*n* = 648	
2.81 (1.83)	3.78 (1.87)	<0.001

SD—standard deviation.

**Table 6:** Reduction of bacteria numbers for biofilms after disinfection under various environmental conditions.

Number of Bacteria (log CFU× cm^−2^)			*p*-Value			
Mean (SD), *n* = 144	Mean (SD), *n* = 144	Mean (SD), *n* = 1008				
**Temperature (°C)**						
4 °C	20 °C	37 °C	*p*	*p* 4–20	*p* 4–37	*p* 20–37
2.85 (0.9)	3.53 (1.03)	3.32 (2.09)	0.045	0.008	0.008	0.229
**pH**						
pH 4	pH 7	pH 9	*p*	*p* 4–7	*p* 4–9	*p* 7–9
0.96 (0.38)	3.1 (1.34)	7.01 (0.73)	<0.001	<0.001	<0.001	<0.001
**Salinity (% NaCl)**						
0%	5%	10%	*p*	*p* 0–5	*p* 0–10	*p* 5–10
3.64 (1.97)	2.74 (0.79)	1.42 (0.55)	<0.001	<0.001	<0.001	<0.001
**Nutrients Availability (BHI)**						
0.5	1	1.5	*p*	*p* 0.5–1	*p* 0.5–1.5	*p* 1–1.5
2.18 (0.75)	3.30 (2.05)	4.32 (0.65)	<0.001	<0.001	<0.001	<0.001

SD—standard deviation.

**Table 7:** Predictive factors for the reduction of bacteria number in biofilm identified by Linear Mixed Effects Model (LMM).

Factor	Value	Std. Error	*t*-Value	*p*-Value
(Intercept)	4.549	0.091	49.801	<0.001
**Disifectant**				
Jodat vs Quatosept	−0.669	0.038	−17.599	<0.001
Peroxat vs Quatosept	−0.712	0.038	−18.73	<0.001
Chlorox S vs Quatosept	−0.274	0.038	−7.198	<0.001
**Disinfectant Concentration**				
Concentration 100 vs 50	0.964	0.027	35.86	<0.001
**Temperature**				
Temp 4 vs. 37	−1.764	0.057	−30.926	<0.001
Temp 20 vs. 37	−1.09	0.057	−19.108	<0.001
**pH**				
pH 4 vs. 7	−3.652	0.057	−64.025	<0.001
pH 9 vs. 7	2.393	0.057	41.961	<0.001
**Salinity**				
Zasol 5 vs. 0	−1.876	0.057	−32.896	<0.001
Zasol 10 vs. 0	−3.196	0.057	−56.036	<0.001
**Nutrients Availability (BHI)**				
Dost 0.5 vs. 1	−2.433	0.057	−42.652	<0.001
Dost 1.5 vs. 1	−0.292	0.057	−5.118	<0.001

**Table 8:** Experimental conditions used in the study.

Changing Environment Parameter	Experimental Conditions	Temperature	pH	Salinity	Nutrient Availability
**Temperature**	1	4 °C	7	0% NaCl	BHI 1.0
	2	20 °C	7	0% NaCl	BHI 1.0
	**3**	**37 °C**	**7**	**0% NaCl**	**BHI 1.0**
**pH**	4	37 °C	4	0% NaCl	BHI 1.0
	**5**	**37 °C**	**7**	**0% NaCl**	**BHI 1.0**
	6	37 °C	9	0% NaCl	BHI 1.0
**Salinity**	**7**	**37 °C**	**7**	**0% NaCl**	**BHI 1.0**
	8	37 °C	7	5% NaCl	BHI 1.0
	9	37 °C	7	10% NaCl	BHI 1.0
**Nutrients availability**	10	37 °C	7	0% NaCl	BHI 0.5 *
	**11**	**37 °C**	**7**	**0% NaCl**	BHI 1.0 *
	12	37 °C	7	0% NaCl	BHI 1.5 *

* BHI 1.0—medium containing amount recommended by the manufacturer, BHI 0.5—medium containing 50% of amount recommended by the manufacturer, BHI 1.5—medium containing 150% of amount recommended by the manufacturer. The control variant was marked with bold font and the variable parameters with gray color.

**Table 9:** Characteristic of disinfectants used in the study.

Disinfectant	Active Substance	Producer	Working Concentration	pH of Solution	
				50% WS *	100% WS
Quatosept	alkyldimethylbenzylammonium chloride	Galvet	2.5 mL/L	6.9	7.2
Peroxat	peractetic acid, hydrogen peroxide	Agro-trade	5 mL/L	3.2	3.4
Jodat	iodine	Agro-trade	5 mL/L	2.9	3.1
Chlorox S	sodium hypochlorite	NTCE	0.7%	10.4	10.1

* WS—work solution.
